# 1881. Costs of Treating Multidrug Resistant Tuberculosis in California, 2022

**DOI:** 10.1093/ofid/ofad500.1709

**Published:** 2023-11-27

**Authors:** Shereen Katrak, Rebecca Wang, Pennan Barry

**Affiliations:** California Department of Public Health, Richmond, California; California Department of Public Health, Richmond, California; California Department of Public Health, Richmond, California

## Abstract

**Background:**

New, shorter regimens containing bedaquiline, pretomanid, and linezolid (BPaL) are entering clinical use for treating multidrug-resistant tuberculosis (MDR TB). Using a micro-costing approach, we estimated the direct cost of individual episodes of care for MDR TB for four different regimens and compared the cost of BPaL regimens with previously recommended, longer regimens.

**Figure:**
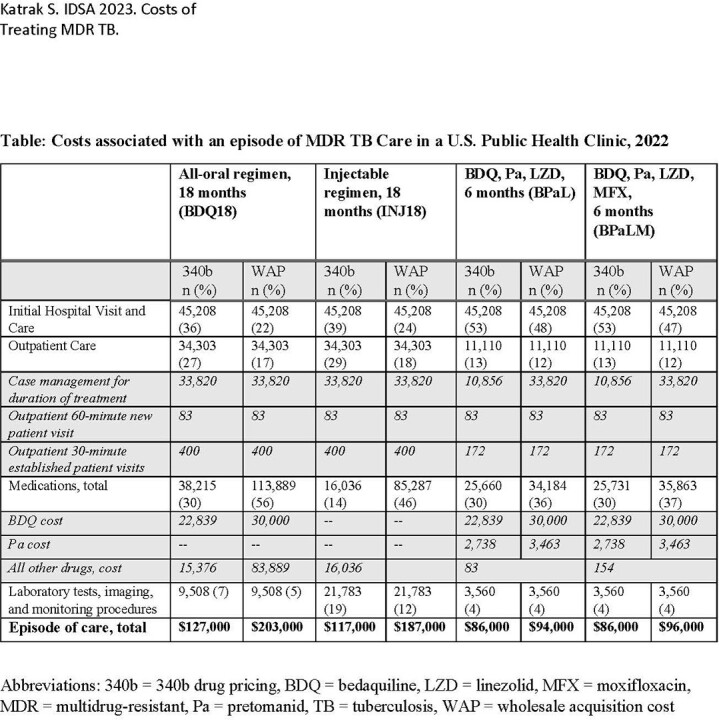

**Methods:**

We estimated the cost of four regimens: an 18-month 5-drug regimen with an injectable agent but no bedaquiline (INJ18), a U.S. guideline-based 18-month 5-drug all oral regimen with bedaquiline (BDQ18), and two 6-month regimens: BPaL and BPaL plus moxifloxacin (BPaLM). We established a standardized set of services for each regimen, assumed no treatment side effects or treatment failure, and summed cost components in 2022 dollars. Cost components included inpatient care costs based on published literature, medication costs (340B and wholesale acquisition prices) paid by California public health TB programs, outpatient laboratory, imaging and physician fees based on California Medicaid reimbursement schedule, and case management (e.g., nurse case manager or outreach worker time, adherence incentives) based on published literature.

**Results:**

Total direct cost for MDR TB care was $86,000-94,000 for BPaL, $86,000-96,000 for BPaLM, $117,000-187,000 for INJ18, and $127,000-203,000 for BDQ18. Hospitalization costs comprised the largest proportion of direct costs for BPaL/BPaM, regardless of type of drug pricing (47-53%) but were also a substantial component of other regimens (22-39% for BDQ18 and INJ18). Bedaquiline was the largest contributor to cost of medications for BPaL and BPaLM even with public health pricing.

**Conclusion:**

MDR TB care is costly. Despite high cost of medications, shorter BPaL/BPaLM regimens may provide cost savings.

**Disclosures:**

**All Authors**: No reported disclosures

